# A comparison of vendor artificial intelligence solutions for automated post-processing of short-axis cine images in cardiovascular magnetic resonance imaging

**DOI:** 10.1038/s41598-026-54182-z

**Published:** 2026-06-02

**Authors:** Thomas Hadler, Clemens Ammann, Hadil Saad, Yashraj Bhoyroo, Jana Veit, Teodora Chitiboi, Jens Wetzl, Christian Geppert, Jeanette Schulz-Menger

**Affiliations:** 1 Charité – Universitätsmedizin Berlin, Corporate Member of Freie Universität Berlin and Humboldt Universität Zu Berlin, ECRC Experimental and Clinical Research Center, Berlin, Germany; 2 Working Group On CMR, Experimental and Clinical Research Center, a cooperation between the Max Delbrück Center for Molecular Medicine in the Helmholtz Association and the Charité – Universitätsmedizin Berlin, Lindenberger Weg 80 13125, Berlin, Germany; 3 DZHK (German Centre for Cardiovascular Research), Berlin, Germany; 4 Department of Cardiology and Nephrology, Helios Hospital Berlin-Buch, Berlin, Germany; 5 Digital Technology and Innovation, Siemens Healthineers AG, Erlangen, Germany; 6 Research & Clinical Translation, Magnetic Resonance, Siemens Healthineers AG, Erlangen, Germany

**Keywords:** CMR image analysis, Artificial intelligence, Machine learning, Automated post-processing, AI comparison, Cardiology, Computational biology and bioinformatics, Diseases, Medical research

## Abstract

Automated segmentation of cardiac magnetic resonance (CMR) imaging is integrated into clinical workflows, yet comparative performance across vendor AI solutions remains insufficiently characterized. This study assessed three models (two commercial, one research) for short-axis cine segmentation in a diverse cohort of 346 cases, including dilated cardiomyopathy (DCM), left ventricular hypertrophy (LVH), healthy volunteers, and other cardiac diseases. Clinical parameter agreement between AI-derived and expert-derived ventricular volumes and left ventricular mass (LVM) was evaluated using correlations and mean differences, segmentation agreement with Dice coefficient, and slice detection was characterized with false positive and negative rates (FPR/FNR). Papillary muscle (PM) inclusion was examined with subgroup analyses. AI-derived clinical parameters agreed strongly with expert measurements (r > 0.8). Nevertheless, inter-model biases included differing ventricular volume estimates. Midventricular segmentation was reliable (Dice > 80%), whereas apical slices were poor (Dice < 65%) with minor area impact (< 1cm^2^). Basal slice detection varied substantially, with AI1 and AI2 over- and AI3 under-detecting slices (e.g. RV FPR: AI1 24%, AI2 14%, AI3 FNR: 32%), producing large area differences. Due to PM exclusion AI2 overestimated volumes and underestimated LVM – particularly LVH-cases. While AI-expert agreement is high, AI solutions are not interchangeable and produce clinically relevant differences to experts across cardiac regions and disease groups.

**Supplementary Information:**

The online version contains supplementary material available at 10.1038/s41598-026-54182-z.

## Introduction

Cardiovascular magnetic resonance (CMR) offers high-resolution cine imaging, acknowledged as the reference for assessing cardiac function and volumes^1,2^. Accurate quantification of these parameters relies on the precise delineation of cardiac structures, particularly the left and right ventricular endocardial borders and the left ventricular epicardium. However, even among expert readers, inter-observer variability in contouring remains impactful, particularly at basal and apical slices^3^. Small differences in segmentation can translate into clinically meaningful changes in derived parameters^4^. Further, post-processing guidelines lack agreement on contouring the papillary muscles^1^. The inclusion of papillary muscles may influence precise volume estimations of pathologies such as left ventricular hypertrophy (LVH) where trabecular inclusion can influence volumetric assessments of the blood pool^5^.

In recent years, the field has witnessed rapid progress in artificial intelligence (AI) methods for automated segmentation^6–8^. These methods are driven by convolutional neural networks, which have demonstrated impressive performance in multiple segmentation challenges, including the Left Ventricle Segmentation Challenge^9^, the Right Ventricle Segmentation Challenge^10^, the Automated Cardiac Diagnosis Challenge (ACDC)^11^, and the Multi-centre, Multi-vendor, and Multi-disease (MnM) challenge^12^. AI models evaluated on such datasets have achieved high segmentation accuracy and derived clinical parameters that often approach the level of expert readers.

Despite this progress, automated contouring is not without limitations. Some AI tools produce anatomically implausible or fragmented contours that violate cardiac geometry constraints^13^, while others may fail entirely on images taken from unseen technical setups (e.g. different vendors), resulting in missing segmentations or inaccurate clinical parameter estimates^14,15^. Nonetheless, building on their success, several AI tools have achieved CE-marking or FDA approval and are now integrated into commercial software platforms used in routine clinical CMR and are already influencing clinical workflows. However, there is little evidence on how the real-world performance of these commercial AI tools perform in comparison to one another or expert readers. In particular, no studies have evaluated how differences in anatomical decisions and segmentation behaviour across vendors affect derived parameters at a clinically meaningful level.

The aim of this study is to evaluate and compare three vendor-supplied AI solutions for automated CMR post-processing, focusing on their ability to generate anatomically plausible segmentations and derive clinically precise parameters across a diverse cohort.

## Methods

### Study population

This study analysed 350 clinical cardiac MRI datasets, representing a broad range of cardiac function and pathology. Based on chart review and image findings (Supplementary Table S1), cases were grouped into four subcategories:

- Healthy: no structural or functional abnormalities
- LVH: patients with left ventricular hypertrophies
- DCM: patients with dilated cardiomyopathies
- Other: cardiovascular diseases, but with normal left-ventricular function

Four cases were excluded from analysis due to multi-slice segmentation failures with nonsensical parameter deviations. These can be inspected as Supplementary Fig. S2 – S5. The remaining cases are summarized in Table 1.

**Table 1:** Study Population. Abbreviations: DCM: dilated cardiomyopathy, LVH: left ventricular hypertrophy

Diagnosis	N [#]	Age [years] (avg ± std)	Sex (M / F / O)
All	346	51.6 ± 17.8	187 / 158 / 1
Healthy	103	38.9 ± 15.5	43 / 60 / 0
LVH	30	60.5 ± 14.9	17 / 13 / 0
DCM	31	49.3 ± 15.1	18 / 13 / 0
Other	182	57.7 ± 15.7	109 / 72 / 1

All cases were acquired on Siemens scanners at varying field strengths (318 at 1.5 T and 28 at 3 T). The average in-plane pixel spacing was 1.79 ± 0.32 mm, and the mean slice thickness was 7.08 ± 0.37 mm. Varying scanner types and acquisition parameters are available as Supplementary Table S6.

### Ethical approval

All MRI datasets (i.e. cases) were anonymized and used in compliance with local ethical standards for retrospective research under the ethical agreement. This was approved by the local ethics committee of the Charité Universitätsmedizin Berlin (study ID: EA 1253 21). All methods were carried out in accordance with relevant guidelines and regulations. The MRI datasets are available from the data protection office at datenschutzbeauftragte@charite.de on reasonable request.

### Ground-truth segmentation

Reference segmentations were provided by a CMR expert with more than five years of experience in clinical CMR interpretation. All contours underwent a consensus review with a senior SCMR Level 3 cardiologist (over 20 years of experience), who provided approval after adjudication of any discrepancies. Cvi42 (version 5.11) was used solely as a manual contouring interface; no AI-assisted or automated segmentation was used for reference generation:

- Left ventricular endocardial contours (LV Endo)
- LV epicardial contours, merged with LV endocardial ones to generate LV myocardial contours (LV Myo)
- Right ventricular endocardial contours (RV Endo)
- LV papillary muscle contours

LV Endo, RV Endo, PM were contoured at end-diastole (ED) and end-systole (ES); LV Myo at ED only.

### AI models evaluated

**Fig. 1:**
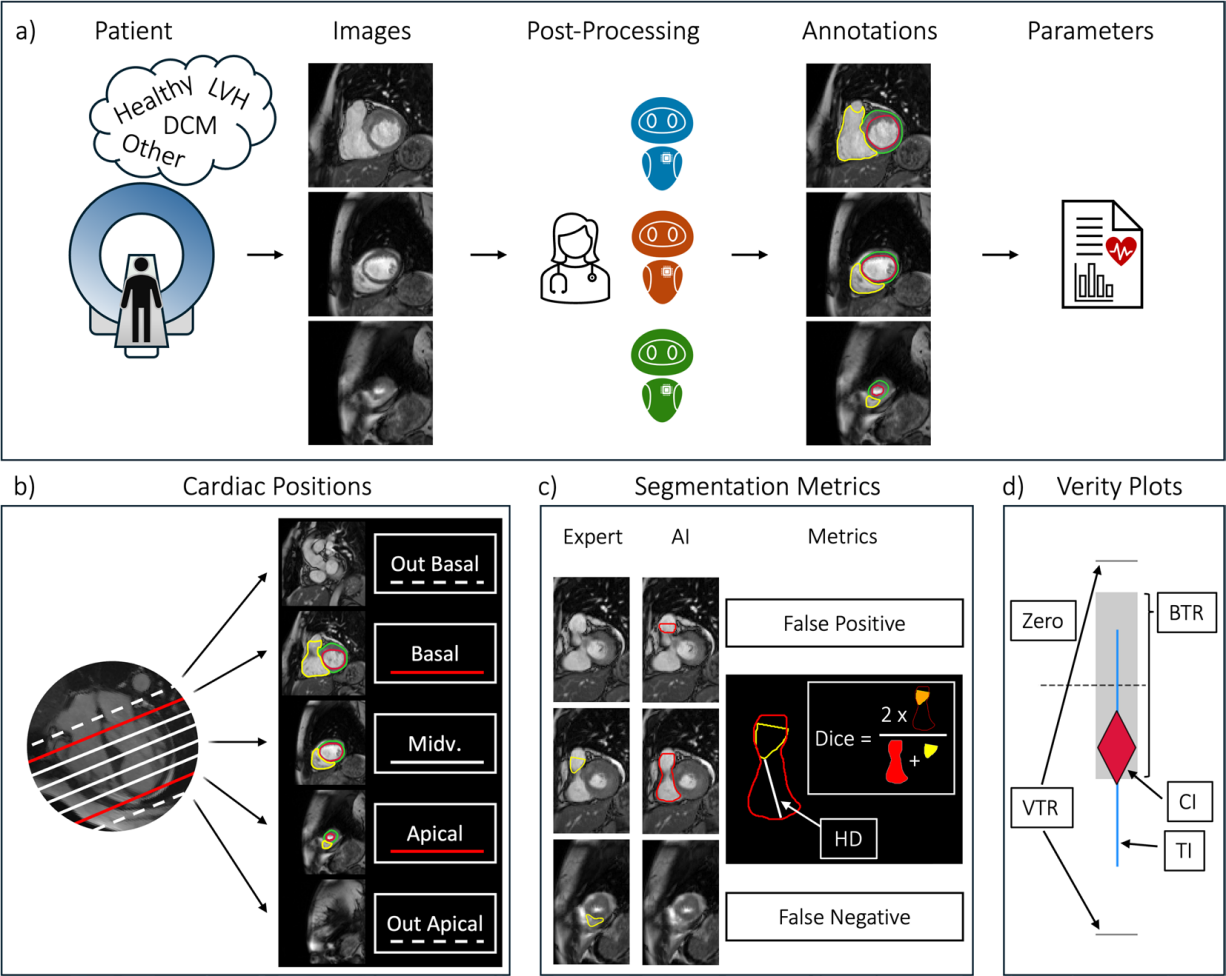
Method Overview. (**a**) Following a short-axis image acquisition, images are annotated by an expert and three vendor AIs. Clinical parameters are derived from the segmentations. (**b**) Cardiac positions are categorized as basal, midventricular, apical, or out-of-scope according to expert-defined anatomical slice inclusion based on SCMR post-processing recommendations. (**c**) Slice-level segmentation decisions are classified (e.g. false positive or negative), and overlap metrics (Dice, Hausdorff Distance) are computed when both expert and AI provide contours. (**d**) Verity plots assess clinical parameter agreement by visualizing whether AI bias (rhombus) and variability (lines) fall within the expert-defined tolerance ranges (BTR and VTR) – red: fail, blue: acceptable. Abbreviations: LVH: left ventricular hypertrophy, DCM: dilated cardiomyopathy, Midv.: midventricular; BTR: bias tolerance range, VTR: variability tolerance range, CI: bias confidence interval, TI: variability tolerance interval.

Three vendor-supplied, AI-based segmentation solutions were evaluated (Fig. 1a):

- CVI42 (version 5.13.7) (Circle Cardiovascular Imaging Inc., Calgary, Canada)
- Medis Suite AI (version 4.0.62.4) (Medis Medical Imaging Systems, Leiden, Netherlands)
- Inline AI research application (Siemens Healthineers AG, Forchheim, Germany)

All model characteristics of the evaluated AI solutions, including differences in deployment and papillary muscle handling, are summarized in Table 2.

**Table 2:** AI Solution Characteristics.

Feature	AI1 (cvi42)	AI2 (Medis suite AI)	AI3 (Siemens research AI)
Type	Commercial	Commercial	Research application
Deployment	Post-processing software	Post-processing software	Inline application
Automation level	Fully automatic	Fully automatic (PM semi-automatic)	Fully automatic
Papillary muscle segmentation	Automatic	Semi-automatic (manual thresholding)	Automatic
Default settings	Yes	Yes	Yes

Each AI model was used with default configuration and no manual corrections applied. The models automatically generated short-axis contours. Both the CVI42 and Siemens Healthineers AI contoured papillary muscles automatically, while the Medis Suite AI offered semi-automatic papillary muscle calculation, requiring manual thresholding. In order to make calculations comparable, papillary muscles were excluded from all calculations, except for the last section, in which the impact of papillary muscles is examined. The calculations with and without PM were performed with dedicated software, Cardiometry – recently presented as an abstract^16^. Cardiometry was re-engineered from Lazy Luna’s backend, a reader comparison tool with years of published applications^17–19^.

### Evaluation metrics

Clinical Parameters (Volume and Function Parameters).

The following functional parameters were calculated for both the LV and the RV: the end-diastolic volume (EDV), the end-systolic volume (ESV), the stroke volume (SV), and the ejection fraction (EF). In addition to this the left ventricular myocardial mass (LVM) was calculated at ED only. AI parameter assessments were compared to expert assessments.

### Verity plots

To jointly assess bias and variability acceptability of each AI model’s output for clinical parameters, Verity plots are used (Fig. 1d)^20^. For each clinical parameter, a confidence interval is computed for the bias, and a tolerance interval for the AI-expert deviations. The confidence interval covers the bias with 95% security; the tolerance interval covers 68% of the AI-expert differences (approx. 1 standard deviation) with 95% security. AI-expert agreement was then defined as the bias confidence interval lying inside a predefined Bias Tolerance Range (BTR), and the tolerance interval lying inside a Variability Tolerance Range (VTR). These tolerance ranges represent expected intra- and inter-reader variability and were derived from 30 cases^20^.

For interpretation, each plot visualizes the distribution of AI-expert differences (violin and swarm plot) alongside the bias estimate (rhombus) and variability (vertical interval lines). An AI model is considered clinically acceptable if the bias estimate lies within the BTR and the variability interval remains within the VTR. Thus, the plot provides a compact visual summary of both systematic deviation (bias) and dispersion (variability) relative to clinically acceptable limits.

### Segmentation accuracy

Three metrics were used to assess contour quality by comparing AI-generated segmentations to ground-truth:

- Dice Similarity Coefficient (Dice): quantifies contour area overlap
- Hausdorff Distance (HD): quantifies contour boundary distance
- Area Differences (relative and absolute): differences in contour-enclosed areas

In addition to this, we calculated segmentation decision metrics to measure when an AI missed or falsely segmented slices. To this end, basal, midventricular and apical slices were defined for the expert and AI models alike (Fig. 1b & 1c):

- Basal: the expert’s most superior segmented slice. For the AIs, all slices segmented above this slice are “basal false positive”, overlooking it is “basal false negative”
- Apical: the expert’s most inferior segmented slice. For the AIs all slices segmented below this slice are “apical false positive”, overlooking it is “apical false negative”
- Midventricular: all slices between the expert’s basal slice and apical slice

The expert selected basal and apical slices according to SCMR post-processing recommendations. Basal slices were included when at least 50% of the ventricular blood pool was surrounded by myocardium, while apical slices were included based on the presence of identifiable ventricular myocardium.

Metrics were computed for:

- LV endocardium (ED, ES)
- LV myocardium (ED only)
- RV endocardium (ED, ES)

### Analysis

The acceptability of vendor-AI clinical parameters is reported for the entire cohort. The acceptability of AI bias and variability is determined with tolerance ranges and presented with Verity plots. AI-expert agreement is quantified with Pearson’s correlation coefficient. For each clinical parameter, AI-expert differences are tested using one-sample t-tests (null hypothesis: bias = 0), and corresponding p-values are reported. A significance threshold of p < 0.05 was used. Normality of the difference distributions was assessed visually via histograms and Q-Q plots. Differences are additionally summarized as mean ± standard deviation.

Segmentation behaviour and choices are investigated as AI-expert contour agreement. False positive and negative rates are calculated to describe segmentation decisions per slice. Dice, HD and area differences are calculated to describe contour agreement. Segmentation metrics are reported as mean deviation, sorted by cardiac position (basal, midventricular, apical) and by contour type (i.e. LV Endo, RV Endo, LV Myo). Following this, example images and segmentations are presented.

To measure the number of required manual corrections, contour-specific cut-offs were defined for short-axis segmentations. These cut-offs represent tolerable deviations at the individual contour level based on human interobserver variability. For this purpose, manual segmentations from 44 cases were compared. A cut-off of ± 1.96 standard deviations of the interobserver difference was defined for volume-based metrics, and a cut-off of the median minus the median absolute deviation was defined for the Dice coefficient.

Clinical parameter acceptability is reported for disease-subgroups, with and without the inclusion of papillary muscles with tolerance ranges via Verity plots. The significance of differences is tested with one-sample t-tests and p-values are reported – without multiple-comparison correction (e.g. Bonferroni or FDR), as the purpose of these tests was descriptive comparison across related performance metrics rather than independent hypothesis testing.

## Results

### Clinical parameters

**Fig. 2:**
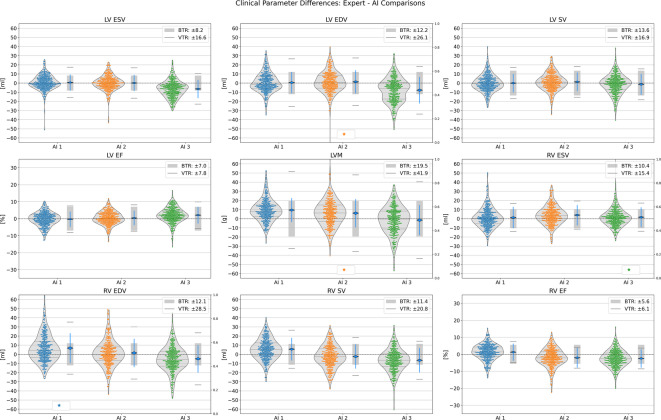
Clinical parameter Differences: Expert – AI Comparisons (Best viewed on monitor). Caption: Each parameter contains three side-by-side Verity plots, one for each AI-Expert comparison. A Verity plot combines a swarm plot (each individual point represents an AI-Expert difference for one case) within a violin plot, to visualize the distribution of differences. On the violin plot’s right side a reliability plot is presented. Each plot’s legend includes the bias and variability tolerance ranges (BTR, VTR). The BTR is shown as the grey area around zero, the VTR is plotted as the two horizontal lines above and below. An AI’s bias is acceptable if the rhombus is contained in the BTR, and the variability is acceptable if the two vertical lines remain within the VTR. Outliers are marked with an *—with the outlier between ± 50 ml and ± 70 ml. Abbreviations: LV: left ventricular, RV: right ventricular, ESV: end-systolic volume, EDV: end-diastolic volume, SV: stroke volume, EF: ejection fraction, LVM: left ventricular myocardial mass, BTR: bias tolerance range, VTR: variability tolerance range.

The distribution of differences, the differences themselves – as well as their acceptability – are illustrated in Fig. 2 with Verity plots. For all AIs and all parameters both the bias and the variability were acceptable when calculated for the entire cohort.

All vendor-AI models demonstrated very strong correlations (r > 0.8) with expert-derived clinical parameters across the full cohort, particularly for LV and RV volume measures (r > 0.9), while correlations for functional parameters (EF and SV) were slightly lower (Table 3).

**Table 3:**
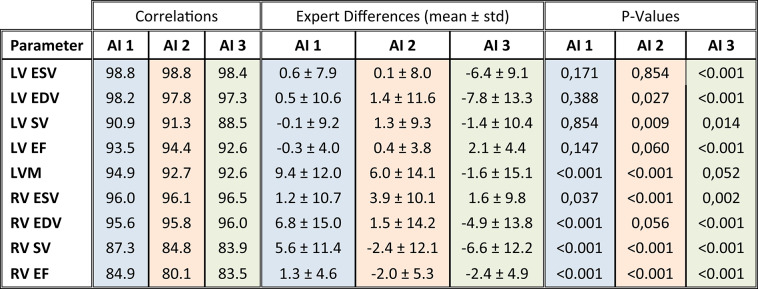
Clinical Parameters: AI-Expert Correlations, Differences and Significance. Abbreviations : LV: left ventricular, RV: right ventricular, ESV: end-systolic volume, EDV: end-diastolic volume, SV: strokevolume, EF: ejection fraction, LVM: left ventricular myocardial mass.

Although clinical parameter assessments were acceptable, they differed between AIs. AI1 and AI2 showed minimal bias for LV volumes (p > 0.05), with mean differences near zero for both EDV and ESV. In contrast, AI3 underestimated LV volumes (e.g. LV EDV bias: -7.8 ml, p < 0.001), though this was offset in derived functional parameters (SV and EF). For LVM, AI3 produced the lowest bias (-1.6 g, p > 0.05), while AI1 and AI2 consistently overestimated LVM (9.4 g, 6.0 g respectively, both p > 0.05). Regarding RV parameters, differences were significant. AI2 slightly overestimated RV ESV. For the RV EDV, AI1 tended to overestimate, and AI3 slightly underestimate these volumes. Despite these volume differences, RV EF estimates were unbiased across all three vendor AIs.

### Segmentation metrics

The metric values for contouring behaviour as well as contouring decisions are summarized in Table 4. We describe the table by cardiac position and AI with notes on contour types when relevant.

**Table 4:**
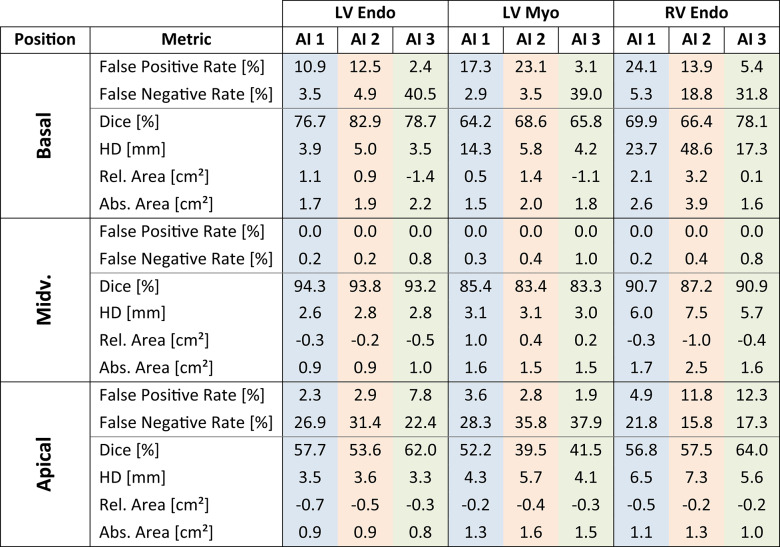
Segmentation Metrics. LV Endo: left ventricular endocardial contour, LV Myo: left ventricular myocardial contour, RV Endo: right ventricular endocardial contour, Midv.: midventricular, HD: Hausdorff distance, Rel.: relative, Abs.: absolute.

**Basal performance**: For AI1 there were high false positives across all structures (e.g. RV Endo: 24%), reflecting over-inclusion, low false negative rates (LV Endo: 3.5%), indicating a bias towards including uncertain anatomy. While AI1 had moderate Dice scores, HD was high for LV Myo and RV Endo (14 mm, 24 mm), suggesting poor basal contour alignment. AI2 behaved similarly, with a comparable pattern of false positives and a tendency to over-include. However, for the RV, AI2 showed a more balanced trade-off between false positive and negative slices. Moreover, Dice scores were slightly better than for AI1 for LV Endo (83%), but the RV HD is extremely poor (49 mm), reflecting the anatomical misplacement of RV Endos. AI3 had very low false positive rates, suggesting conservative segmentation decisions, with very high false negative rates (e.g. LV Endo: 40.5%). When slices were included, AI3 had the best HD and Dice values. Differences are clear: AI1 and AI2 favour inclusion, AI3 favours exclusion, AI2 is less stable in RV geometry.

**Midventricular performance**: AI1 had the highest Dice scores (LV Endo: 94%, LV Myo: 85%, RV Endo: 91%) and good HD values across contours. No false positives, hardly any false negatives. AI2 had slightly lower, but still strong Dice values (LV Endo: 94%, LV Myo: 83%, RV Endo: 88%) and good HD values, with a slightly higher HD for RV Endo. No false positives, hardly any false negatives. AI3 had slightly lower Dice values than AI1, similar to AI2. It had the lowest HD overall, especially for RV, indicating a high shape accuracy.

**Apical performance**: AI1 had moderate false negative rates (22%—27%) and low false positives (2%—5%) and moderate Dice scores. AI2 had slightly higher false negative rates than AI1 (16%—36%), with similar false positive rates, except for the RV Endo (12%). AI3 had false negative rates between AI1 and AI2 (17%—38%) with false positive rates similar to AI2.

**Fig. 3:**
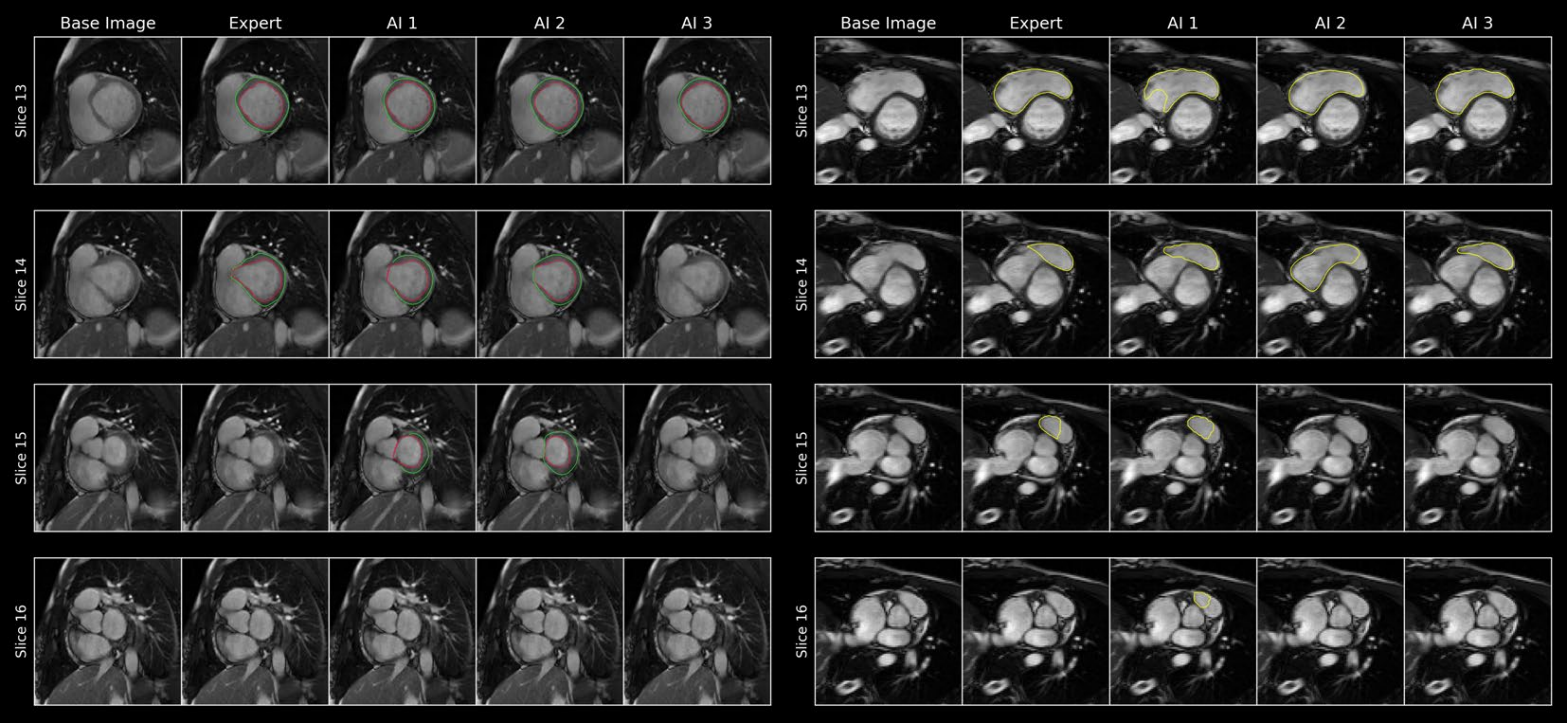
Segmentation Decision Failures (Best viewed on monitor). Caption: For two cases (left and right), four slices from mid ventricle to base are presented. The left case focusses on typical segmentation decision failures for the left ventricle, where AI3 overlooked the base as defined by the expert, and AI1 and AI2 produced false segmentations above the expert-defined base (slice 14). The right case focusses on right ventricular failures: AI1 produced a false segmentation above the base (slice 15); AI2 and AI3 missed it.

For all contour types slices were missed or falsely segmented in the basal and apical slices, often causing large area impacts in the basal region. These segmentation decision differences varied between vendor AIs and the expert, such that AIs may start to segment basal / apical slices earlier (false positive) or later than the expert (false negative). Typical basal segmentation decision mistakes for the vendor AIs are shown in Fig. 3.

### Required manual corrections

**Fig. 4:**
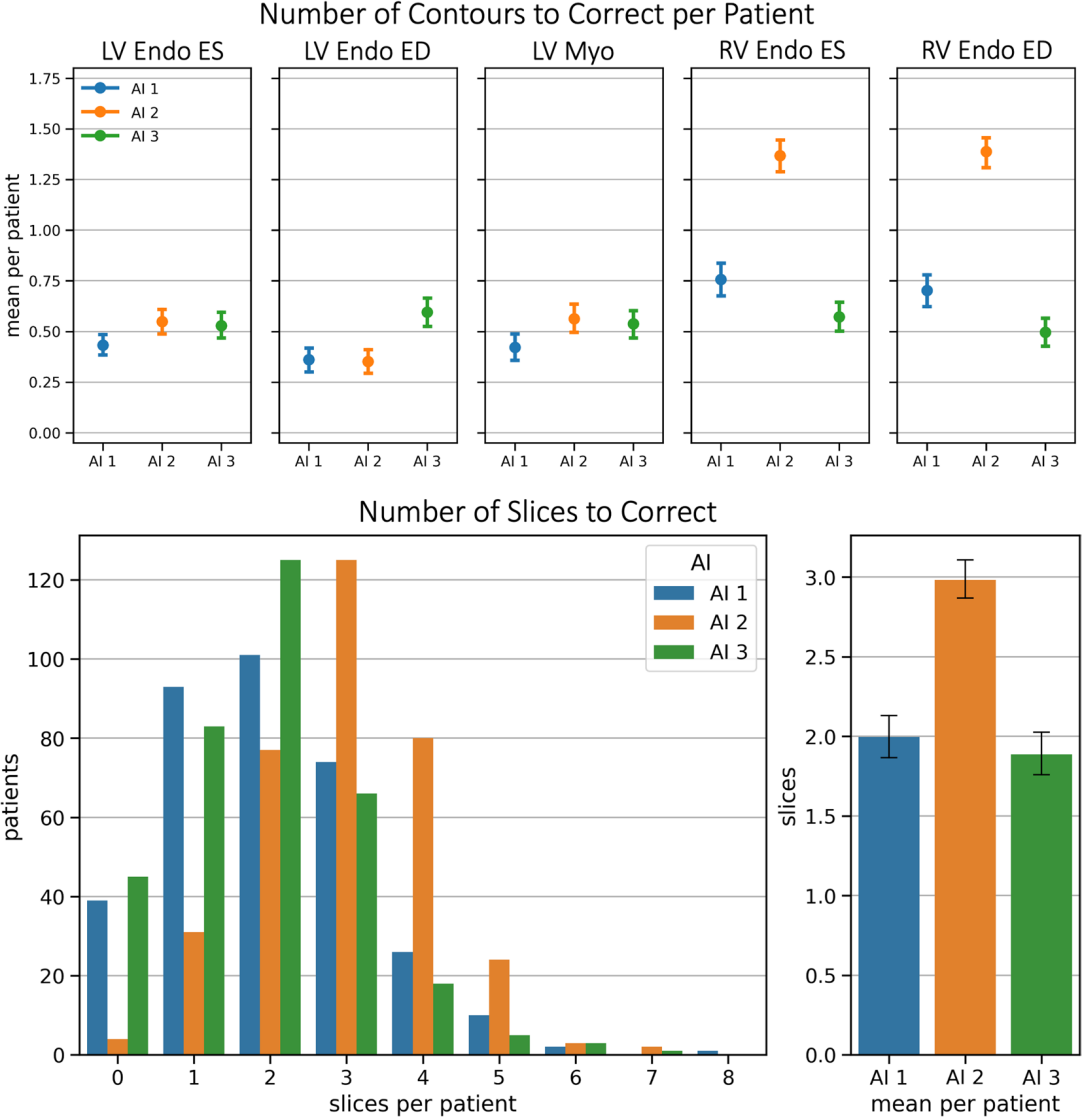
Contours and Slices requiring Correction. The top shows the average number of contours requiring correction per patient, for each contour type. Below, the number of slices requiring correction are illustrated – that is each slices for which at least one contour must be corrected.

Per-slice tolerance intervals were defined as follows: <  ± 5.6cm^2^ and > 90% Dice for LV Endo ED; <  ± 4.0cm^2^ and > 83% Dice for LV Endo ES ; <  ± 5.0cm^2^ and > 71% Dice for LV Myo; <  ± 7.4cm^2^ and > 82% Dice for RV Endo ED; and <  ± 5.5cm^2^ and > 74% Dice for RV Endo ES. The mean number of contours requiring correction per patient was below 1.5 for all contour types and groups (Fig. 4—top). When corrections are considered for any contour (logical OR), on average fewer than three slices per patient need to be corrected in a short-axis stack (Fig. 4—bottom). AI2 required 0.5 more slice corrections in the RV than AI1 and AI3, as well as one more slice correction per case.

### Impact of papillary muscles

**Fig. 5:**
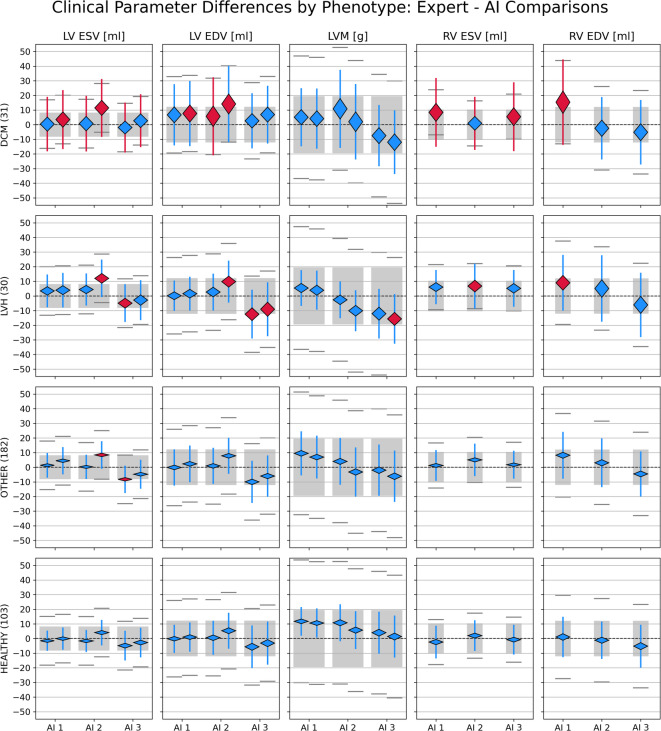
Clinical Parameter Verity Plots by Disease Phenotype – With and Without Papillary Muscles (Best viewed on monitor). Each panel shows a combination of clinical parameter and disease for three vendor AIs. For LV parameters the papillary muscles were excluded in the left Verity plot and included in the right one. Note that this also affects the LVM, as papillary mass is included in the LV mass calculation. Verity plot: the bias’s tolerance range (BTR) is shown as a grey area around zero, the variability tolerance range (VTR) is plotted as the two horizontal lines above and below. An AI’s bias is acceptable if the rhombus is contained in the BTR, and the variability is acceptable if the two vertical lines remain within the VTR. Abbreviations: LV: left ventricle, RV: right ventricle, ESV: end-systolic volume, EDV: end-diastolic volume, LVM: left ventricular mass, DCM: dilated cardiomyopathy, LVH: left ventricular hypertrophy.

Only two AIs contoured the papillary muscles; AI2 did not. The bias and variability intervals are considered within or outside tolerance – visualized in Verity plots (Fig. 5). The plots are displayed for all subgroups with and without papillary muscles. Full statistical details (including function parameters) are available as Supplementary Table S7.

**AI1** parameters were within tolerance for healthy volunteers and other patients (without LVH and DCM). For LVH patients, LV parameters and LVM were within tolerance, regardless of PM inclusion. The RV EDV bias and RV EF variability were outside tolerance (RV EDV bias: 9 ml, p < 0.05, RV EF std: 4.8%). In DCM, the variability of LV ESV exceeded tolerance regardless of PM inclusion (LV ESV std: 13.8 ml no PM, 15 ml with PM). LV volume biases increased with PM inclusion (LV ESV bias: 0.4 ml no PM, 3.5 ml with PM, LV EDV bias: 6.7 ml no PM, 7.6 with PM). However, these compensated to produce within-tolerance LV function and mass (LV SV bias: 6.3 ml p < 0.05 no PM, 4.1 ml p > 0.05 with PM).

**AI2** parameters remained within tolerance for healthy volunteers and other patients when PMs were excluded. However, with PM inclusion, LV ESV bias exceeded tolerance in the Other subgroup (bias: 8.4 ml, p < 0.001). For LVH, LV volume and EF biases exceeded thresholds with PMs included (LV ESV bias: 12 ml p < 0.05, LV EDV bias: 9.8 ml p > 0.05, LV EF bias: -5.7 p < 0.05). RV ESV bias and RV EF variability exceeded tolerance (RV ESV bias: 6.7 ml p < 0.001, RV EF std: 5.9%). In DCM, LV volume variability and LV EDV bias were outside tolerance when PM was excluded. When PM was included, biases and variabilities exceeded tolerance (e.g. LV ESV bias: 0.7 ml no PM to 11.4 ml with PM, LV EDV: 5.7 ml no PM to 14.1 ml with PM). LV function parameters and mass were outside tolerance. RV parameter biases were within tolerance (e.g. RV EF bias: -1.3%, p > 0.05).

**AI3** parameters were generally within tolerance for healthy volunteers and other patients, though LV ESV bias exceeded limits for other patients when PM were excluded (LV ESV bias: -8.3 ml p < 0.05). In LVH, LV ESV bias was outside tolerance when PM was excluded, while LV EDV bias remained outside regardless of PM inclusion (LV ESV bias: from -4.9 ml no PM to -2.8 ml with PM, LV EDV bias: -12.5 ml no PM to -9 ml with PM). LVM exceeded tolerance with PMs included (LVM bias: 15.6 g). While RV volumes within tolerance, RV SV and EF biases showed out-of-range values. In DCM, LV ESV variability was outside tolerance (LV ESV std: 10 ml), while other LV parameters stayed within. RV ESV, SV and EF bias and variability were outside tolerance (e.g. RV EF bias: -3.7 ml, p > 0.05).

## Discussion

All three vendor AI models demonstrated strong performance in clinical parameter estimation, showing high correlation with expert values and largely acceptable bias and variability across most metrics. Our findings further demonstrate that none of the AI models has a consistent advantage over the others. Our analysis reveals substantial differences in how these models operate, particularly in the details of contour generation and anatomic interpretation. They varied in their treatment of basal and apical slice inclusion – a critical step in volumetric estimation. AI1 and AI2 frequently segmented slices above the expert’s most basal slice, whereas AI3 more often excluded valid basal slices. In addition, the models differed in how they handled papillary muscle segmentation with large impacts on expert LV parameter agreement. Model performance also varied with disease status, indicating that underlying cardiac morphology influenced AI reliability. These nuanced differences – often masked when only parameters or overall metrics are reported – highlight the importance of detailed validation before use in clinical routine.

The evaluated AI solutions were selected to represent vendor-integrated workflows currently accessible within our clinical environment, including both commercial and research implementations. In this context, the purpose of this study was not to provide an exhaustive benchmark of all available CMR segmentation tools, but rather to investigate whether clinically relevant differences exist between commonly used AI-assisted workflows. The observed differences in contouring behaviour and anatomical interpretation suggest that vendor-specific validation remains important before clinical deployment.

### Segmentation behaviour and decisions

In general, vendor AI models provide good segmentations and many behaviours were vendor-independent (e.g. excellent midventricular slices or boundary issues close to infolding artefacts). Examples of such good and bad segmentations are available in Supplementary Figure S6. However, segmentation appears to be more difficult in basal and apical slices than for midventricular slices, and more difficult in the LV than in the RV. No AI-solution was superior in every regard, rather interesting differences were revealed in segmentation behaviour and especially in segmentation decisions, in which impactful volume differences are most frequent.

Multi-reader datasets, such as the SCMR Consensus Contour dataset, have demonstrated that even expert-derived LV contours exhibit systematic variability between centres, primarily due to differences in contouring protocols. Notably, the largest disagreements occur at the apex and base, although their impact on volumetric measurements may be smaller than consistent contour placement differences across the ventricle. In this context, our findings extend these observations by showing that vendor AI models exhibit similarly distinct and systematic segmentation behaviours, particularly at the basal and apical boundaries of the short-axis stack.

Basal slice segmentation remains a persistent challenge in both manual and automated approaches, and our findings align with existing literature on its clinical relevance. Prior inter-observer studies have shown that even among experts, basal slice decisions introduce statistically significant variability in LV and RV parameters, with the RV particularly affected due to its complex basal anatomy and unclear valve-plane boundaries^21,22^. This issue is compounded in AI models, where definitions of basal inclusion vary across training datasets and competitions, as noted by Chen et al.^23^. In our study, AI models exhibited distinct strategies for handling basal slices. AI1 and AI2 favoured inclusion, with relatively low false negative rates but elevated false positive rates, especially in the RV – a trade-off that can lead to overestimation of volume and mass. In contrast, AI3 adopted a more conservative strategy, with the lowest false positive rates but the highest false negative slice rates, particularly for LV endo- and myocardial contours, contributing to systematic underestimation of volume and mass. Notably, AI2 demonstrated particular instability in RV geometry at the basal level, with large shape errors (HD) and inconsistent segmentation, suggesting poor generalization in this anatomically variable region. While some studies have reported improved reproducibility when basal inclusion follows guideline-based definitions^24^, such approaches often depend on long-axis images for anatomical reference, which may themselves be misaligned due to inter-breath-hold variability or patient motion.

At the midventricular level, where the stable anatomy is unambiguous, such that all three AI models performed consistently well, with high Dice scores and minimal false positive or negative rates. This cardiac region is where the AI models were most interchangeable. This reflects the greater consensus typically seen among expert readers in this region^25^ and supports previous findings that AI models perform best in midventricular slices^13^. However, subtle differences remain. Notably, AI1 showed a tendency to systematically over-segment the left ventricular myocardium. Minor deviations may accumulate over the stack, leading to clinically relevant overestimations of LV mass. AI2 and AI3 showed better balance in this regard, with more symmetric area differences. Given the widespread use of LV mass in both diagnosis and risk stratification – particularly in hypertrophic cardiomyopathy –even mid-slice segmentation biases warrant careful attention in automated tools. AI2 further underestimated the RV in the midventricular slices, which compensated the basal slice overestimations.

Apical slice segmentation presented the poorest overall performance, with all models missing more than a fifth of all slices, and Dice coefficients consistently below 65%. This mirrors the difficulty experts report in defining the position of the apex, largely due to the small size of apical structures, thin myocardial walls accompanied by partial volume effects, and variable geometry – particularly of the RV.

### Papillary muscles

Papillary muscles are critical for LV quantification^26^, particularly in patients with LVH or DCM^5^. AI2 did not segment PMs, which led to discrepancies when PMs were included in expert contours. Under these conditions, AI2 failed across multiple parameters, including LV volumes, EF, and mass – despite otherwise clean contours. This is not criticism of the AI2’s vendor: they offer semi-automated papillary muscle segmentation via thresholding, which were not included because they could not be bulk-exported – but would be available on a per patient basis. The PM impact analysis demonstrates that PM inclusion provides structurally and functionally relevant information that cannot be ignored in diseased populations emphasizing the importance of consistent methods. While Riffel et al. showed that PM inclusion generally leads to significant parameter differences^26^, Gommans et al. additionally demonstrated that PM inclusion is relevant to parameter averages in LVH patients, particularly for LVM and LV EF^5^.

At the same time, the analysis reveals the challenge of accurate PM segmentation. AI1 and AI3 include papillary muscles, yet both show model- and disease-specific limitations. AI1 performs reasonably well in LVH patients, but its LVM bias approaches tolerance limits and fails in DCM cases when PMs are included. AI3, in contrast, handles DCM cases robustly regardless of PM status, but struggles in LVH patients, where PM inclusion improves volume estimates at the expense of LVM accuracy. These findings emphasize that PM handling is both clinically significant and technically challenging, and that model-specific behaviour must be understood in the context of underlying disease morphology.

### Volumes and function: parameter compensation across phases

Despite this, errors at ED and ES phases often compensated in the calculation of stroke volume and ejection fraction, leading to stable functional outputs. The stability of function parameters has also been noted in a study assessing AI models for real-time CMR, where despite differences in LV ESV and EDV volumes, the EF remained stable^27^. This phase-level compensation does not contradict the impact of less stable volume or LVM assessments.

### Quality assurance and manual correction

While the analysis focused on successfully processed cases, several AI models failed on a small number of challenging studies. These included a case with a large intracavitary thrombus, an LVH case with very large papillary muscles, and two for which otherwise good midventricular slices were overlooked (Supplementary Fig. S2 – S5). Such failures highlight persistent issues with robustness, especially when AI models encounter unseen anatomical presentations or undergo shifts in pixel value distributions. In these cases, models either skipped several midventricular slices or returned no usable segmentation at all. Although excluded from the statistical analysis, these cases are clinically meaningful and illustrate the need for quality control mechanisms integrated into AI-driven workflows.

The most impactful AI-failures were observed in basal slices. Basal slice mistakes can cause significant volumetric deviations due to their disproportionate effect on chamber volumes. These errors are often driven by partial volume artifacts, where a basal image may contain overlapping structures, including valves, atria, or adjacent myocardial tissue, making it difficult to determine whether the region should be included in the analysis (24). Experts often resolve such ambiguities by integrating information from surrounding slices, reviewing the full cine sequence, and consulting long-axis images that provide orthogonal views with a high resolution along the SAX stack’s z-axis. However, these decisions are not always reproducible, particularly when short- and long-axis images are misaligned, which is common, as the sequences are acquired at different time points during the exam and are vulnerable to patient movement.

Despite these challenges, the number of required manual corrections per patient was low: on average, fewer than three slices per case needed correction across the short-axis stack. AI1 and AI3 showed lower correction burdens overall, while AI2 required approximately one additional correction per case and more frequent RV adjustments. This aligns with the HD results, which showed that AI3 produced RV contours most closely matching the expert, while AI1 performed slightly better for the LV endocardium.

### Outlook

Future developments in AI for cardiac MRI must account for the growing diversity of image types and clinical use cases, including parametric mapping techniques, late gadolinium enhancement, and strain calculations. To support meaningful benchmarking, large and more representative challenges should be designed than those often tailored to computer vision groups – not only to assess segmentation accuracy but to reflect real-world clinical utility across disease types, scanner vendors, and anatomical variations. While segmentation challenges have propelled the field forward, commercial vendors remain essential for translating these advances into clinical practice and should be included in future benchmarks.

### Limitations

Although the study builds on 346 cases, only 31 DCM and 30 LVH patients were included. AI solutions are often retrained and may differ between versions, which means that this study is only a snapshot of a vendor AI’s performance. Further, the evaluated vendor solutions represent a selected subset of available CMR AI tools and therefore findings may not generalize to all commercial or research implementations. Use of a single software platform for manual contour generation may introduce subtle platform-dependent influences, although the reference standard was fully independent from the evaluated AI algorithms.

## Conclusion

This study systematically compared three vendor-supplied AI solutions for short-axis cine segmentation in a diverse cohort of 346 CMR cases. While all AIs performed well overall, they differed in basal slice inclusion, papillary muscle segmentation, and disease-specific challenges. These differences can be broadly summarized as extension-prone behaviour for basal slices in AI1 and AI2, and truncation-prone behaviour in AI3, with additional variability in RV contour stability. These differences propagated from anatomical decisions to clinical parameters, underscoring the need for careful validation of AI tools prior to deployment. Our findings highlight that current AIs are not interchangeable and that segmentation behaviour must be interpreted in light of both structural imaging limitations and evolving model architectures. As AI integration into CMR continues, ongoing benchmarking and transparency will be essential to maintain clinical reliability.

## Supplementary Information

Below is the link to the electronic supplementary material.

Supplementary Material 1

Supplementary Material 2

Supplementary Material 3

Supplementary Material 4

Supplementary Material 5

Supplementary Material 6

Supplementary Material 7

Supplementary Material 8

## Data Availability

The MRI datasets analyzed in this study contain patient information and cannot be made publicly available under German data protection regulations. The requirement for written informed consent was acquired during the original clinical studies and was therefore waived in this study due to its retrospective design as approved by the local ethics committee of the Charité Universitätsmedizin Berlin (study ID: EA 1 253 21). Data may be obtained from the Charité data protection office (datenschutzbeauftragte@charite.de).
