# Supplementary figures and images for: A comparison of vendor artificial intelligence solutions for automated post-processing of short-axis cine images in cardiovascular magnetic resonance imaging
Source: Sci Rep. 2026 Jun 2;16:17154. doi: 10.1038/s41598-026-54182-z (PMC13234427; doi:10.1038/s41598-026-54182-z)

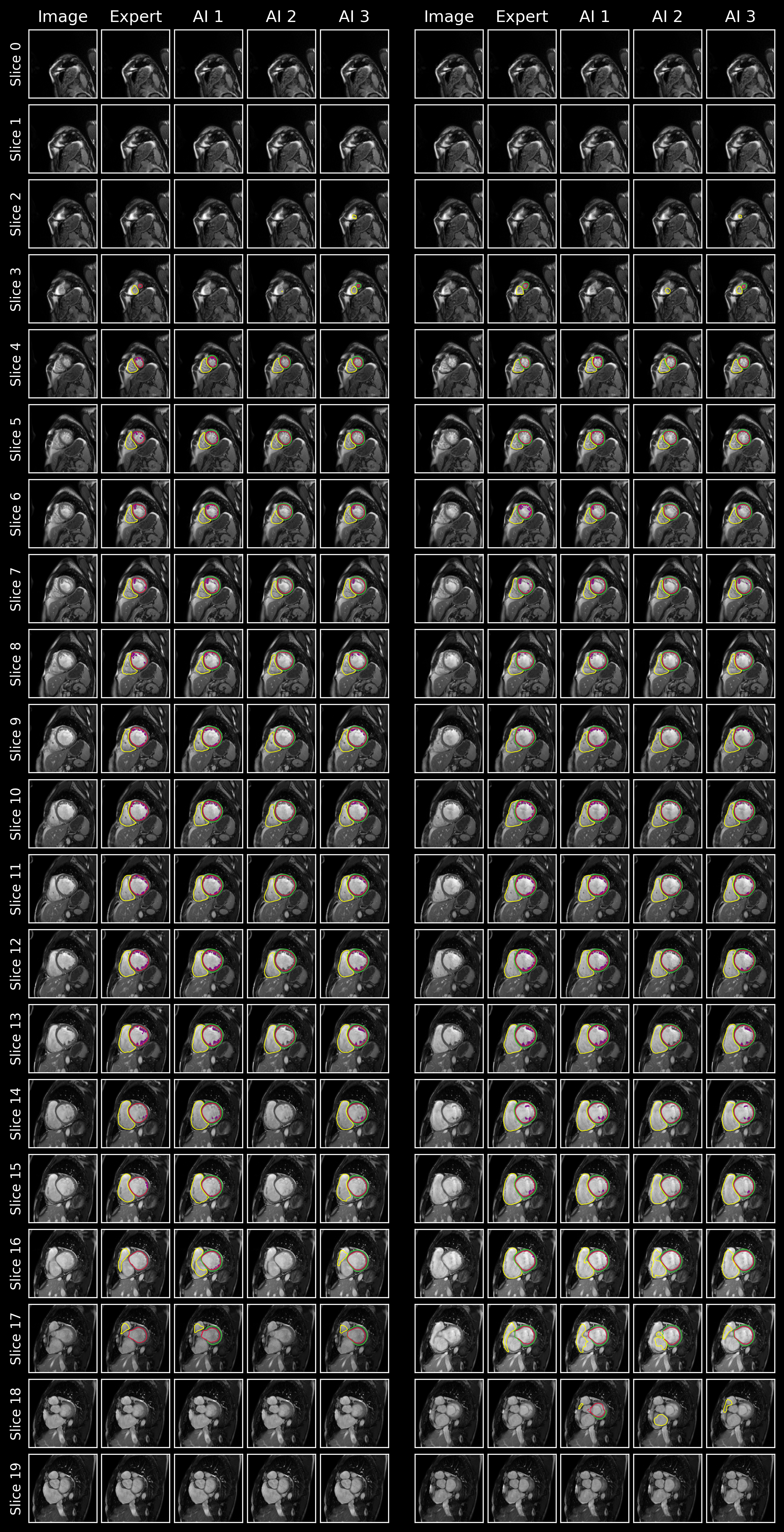

Supplement: Supplementary file 4 — Supplementary Material 4 [file 41598_2026_54182_MOESM4_ESM.png]

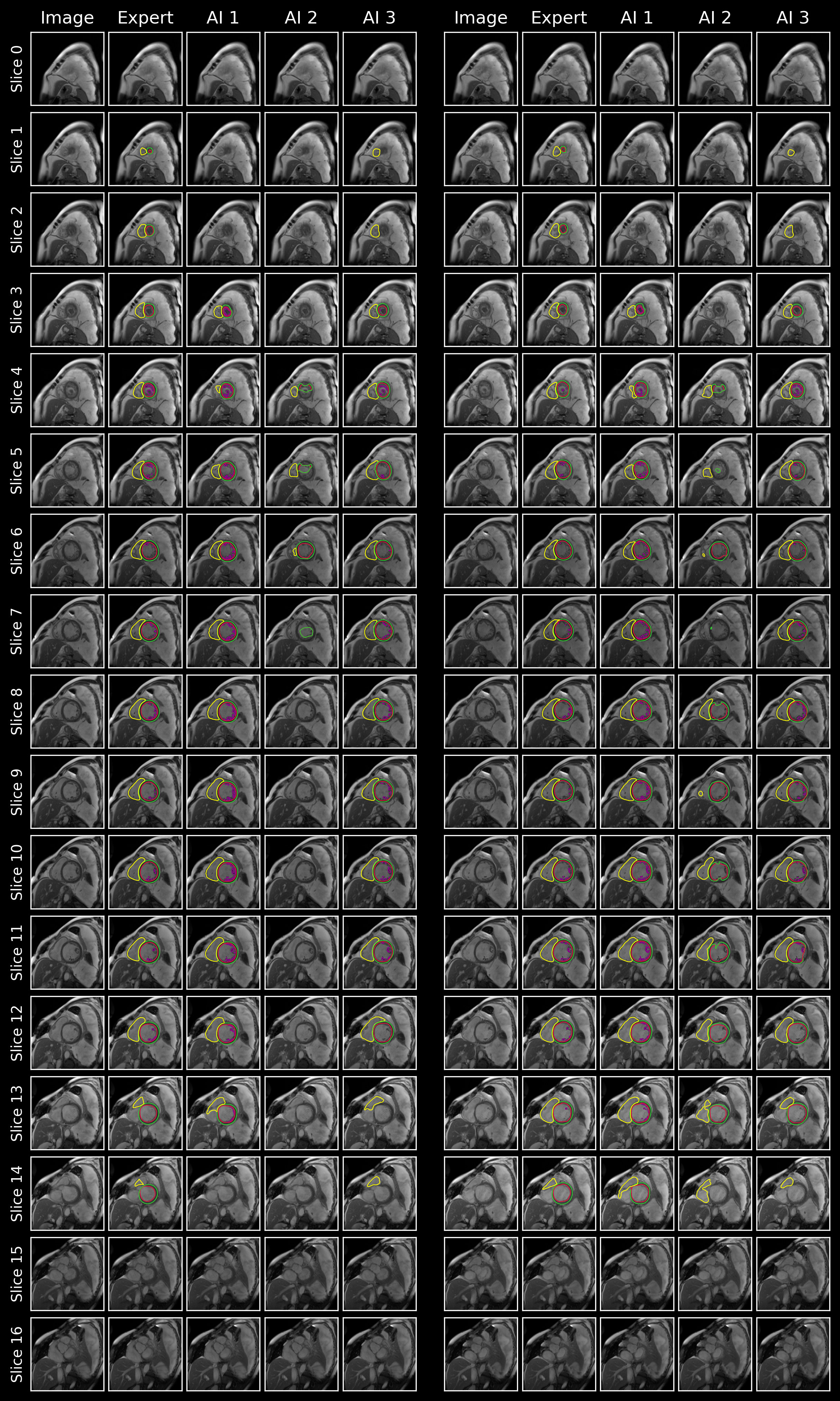

Supplement: Supplementary file 5 — Supplementary Material 5 [file 41598_2026_54182_MOESM5_ESM.png]

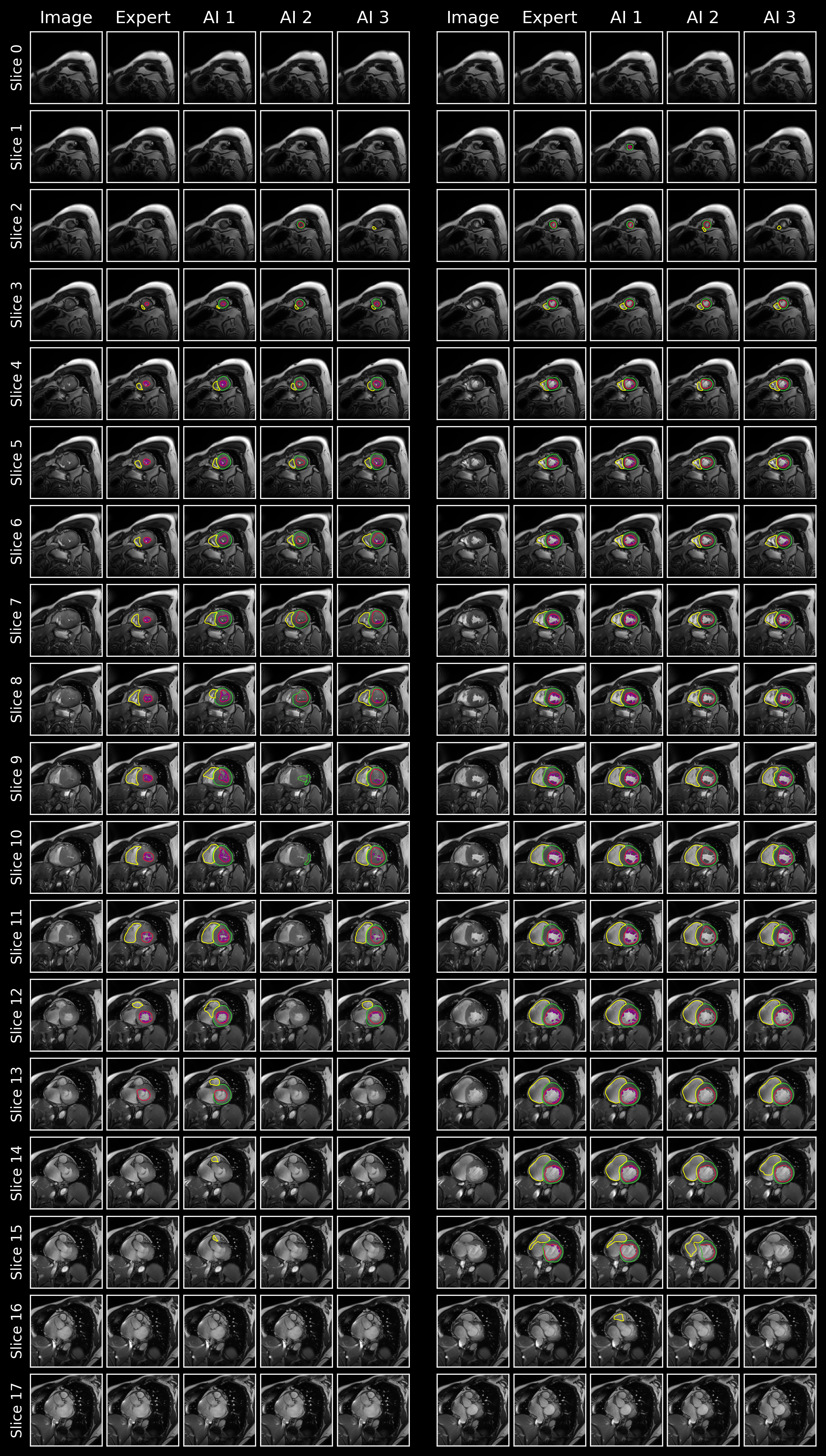

Supplement: Supplementary file 6 — Supplementary Material 6 [file 41598_2026_54182_MOESM6_ESM.png]

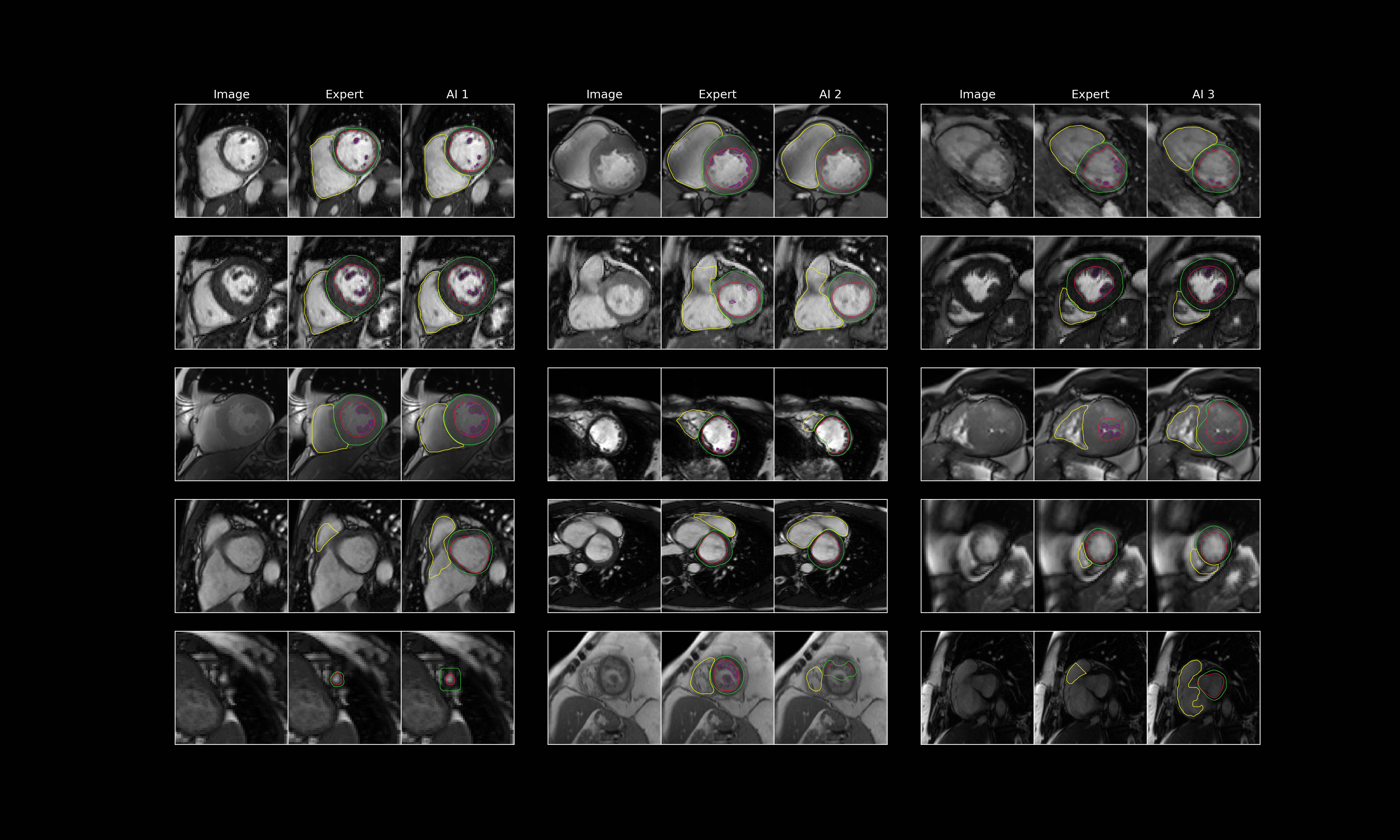

Supplement: Supplementary file 7 — Supplementary Material 7 [file 41598_2026_54182_MOESM7_ESM.png]

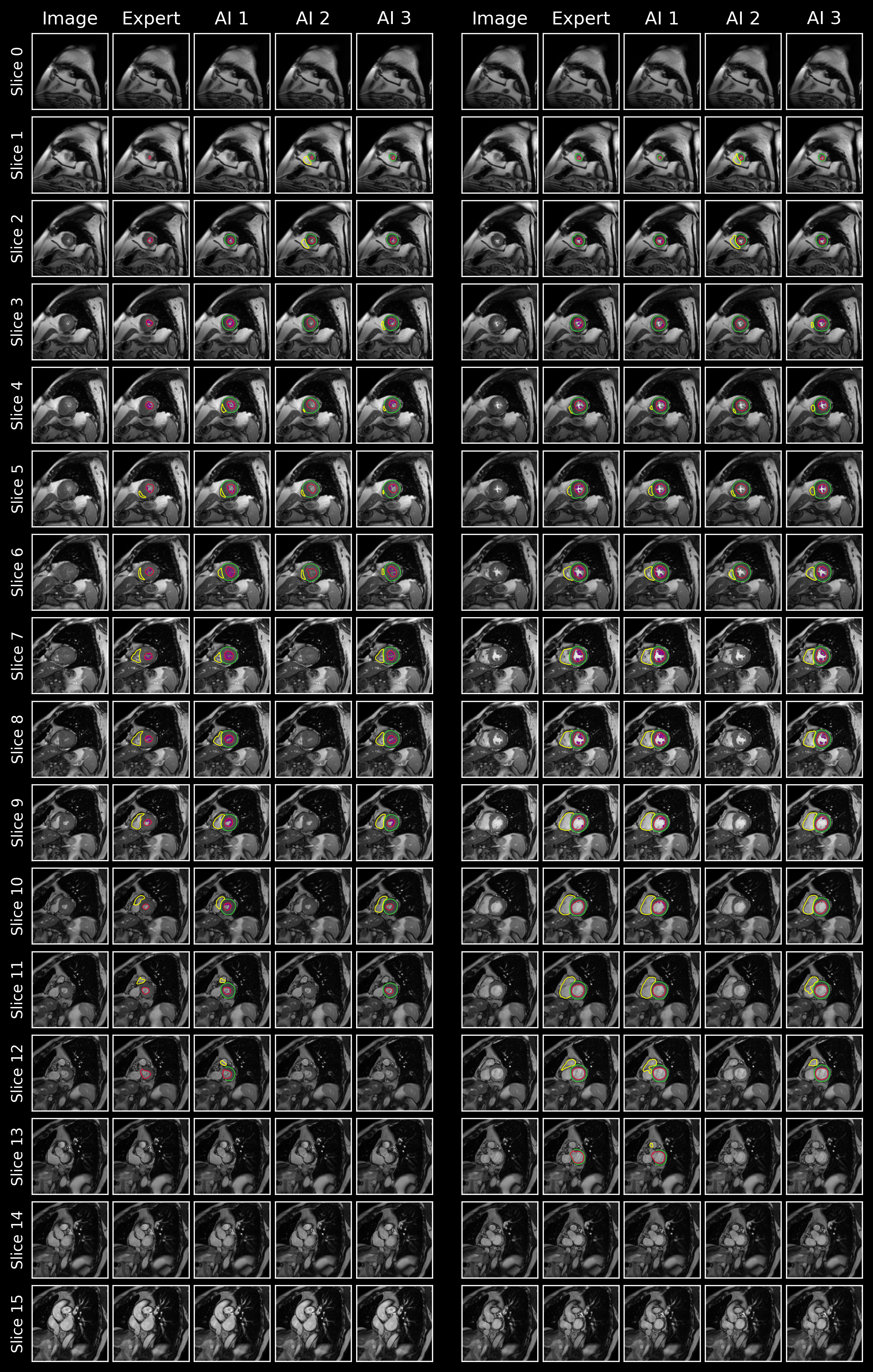

Supplement: Supplementary file 8 — Supplementary Material 8 [file 41598_2026_54182_MOESM8_ESM.png]
